# Development of a Control System for a Hydraulic Injection Molding Machine Using an AFC Controller and Utilization of Learning Parameters

**DOI:** 10.3390/polym18080911

**Published:** 2026-04-08

**Authors:** Takahiro Shinpuku, Takumi Kobayashi, Shota Yabui, Kento Fujita, Yusuke Uematsu, Shota Suzuki, Yusuke Uchiyama

**Affiliations:** 1 Mechanical Systems Engineering, Tokyo City University, Tokyo 158-8557, Japan; g2481030@tcu.ac.jp (T.S.); g2481021@tcu.ac.jp (T.K.); fujitak@tcu.ac.jp (K.F.); 2 Research and Development Center, MAZIN Inc., Tokyo 111-0035, Japan; uematsu@mazin.tech (Y.U.); suzuki@mazin.tech (S.S.); uchiyama@mazin.tech (Y.U.)

**Keywords:** hydraulic injection molding machine, adaptive feedforward control, shot-to-shot learning, data-driven control, process state indicator

## Abstract

Maintaining stable molding quality in hydraulic injection molding machines is difficult because the internal state of molten resin cannot be directly observed and varies with material properties and operating conditions. This difficulty is intensified by variations in hydraulic characteristics caused by oil temperature changes. This study proposes an adaptive feedforward control (AFC) framework that improves injection velocity tracking while utilizing AFC learning parameters as indicators of resin state. AFC is implemented as a multi-frequency feedforward controller whose parameters are updated through repetitive injection cycles. To overcome the limited learning duration within a single injection shot, a shot-to-shot compensation mechanism accumulates and transfers learning results across consecutive shots. Experiments are conducted on a hydraulic injection molding machine using polypropylene materials with different viscosities. The results show that the converged AFC learning parameters vary systematically with material changes and correspond to differences in molded product appearance. Furthermore, by adjusting the cylinder temperature of another material, the AFC parameters converge to values close to those of a reference material, resulting in similar molded products. These findings demonstrate that AFC learning parameters reflect variations in resin state and can serve as practical state indicators for aligning molding conditions.

## 1. Introduction

Injection molding machines are widely used in the plastics industry. They play a central role in plastic manufacturing. They are essential production systems in a wide range of industrial fields, including automotive, electronics, and medical applications. In Japan, injection molding machines account for more than 80% of plastic molding equipment [1]. This indicates their overwhelming presence in industrial applications. In recent years, recycled materials have increasingly been used in plastic products [2,3]. As a result, material properties often vary between production lots. Even under such conditions, high quality, stability, and repeatability are strongly demanded in industrial injection molding. Molding quality is strongly influenced by the internal state of molten resin, particularly its viscosity [4]. Therefore, understanding resin state variations during the injection process and reflecting them in control is essential for achieving stable molding quality.

### 1.1. Difficulty in Observing Resin State

In injection molding machines, directly observing the internal state of molten resin during operation is generally difficult [5]. Recently, AI-based in-line quality = 0monitoring methods have been studied [6]. However, such approaches evaluate product quality after molding rather than directly reflecting the internal process state during injection. In practical industrial systems, only limited sensor information is available for control and monitoring. Typical measurable signals include screw position, injection velocity, and hydraulic pressure. However, key resin properties such as viscosity and flow behavior cannot be directly measured during the injection process. These properties vary dynamically depending on material characteristics, temperature, and operating conditions. As a result, it is difficult to quantitatively evaluate resin state variations in real time. In industrial practice, molding conditions are often adjusted based on indirect indicators and operator experience [7]. Such adjustments rely heavily on trial-and-error procedures. When material properties vary between production lots, this approach becomes increasingly inefficient. Moreover, variations in material properties can significantly increase the variability of resin state during injection. Such variations arise from differences in material batches, thermal conditions, and processing history. This increased variability makes it difficult to maintain stable molding quality using fixed molding conditions. Therefore, the lack of direct and quantitative information on resin state remains a fundamental challenge in injection molding processes.

### 1.2. Resin State Estimation Challenges in Hydraulic Injection Molding

The difficulty in observing resin state becomes more pronounced in hydraulic injection molding machines. Compared with electric injection molding machines, hydraulic systems exhibit additional sources of uncertainty. In electric injection molding machines, several studies have reported indirect estimation of resin pressure or viscosity using motor torque information [8,9]. Since the motor directly drives the screw, torque signals can be used as indicators of load changes caused by resin flow behavior. In contrast, hydraulic injection molding machines transmit driving force through hydraulic oil. As a result, the relationship between actuator input and screw motion is strongly affected by hydraulic characteristics. These characteristics vary depending on oil temperature, pressure losses, and mechanical friction. In particular, changes in oil temperature significantly alter hydraulic viscosity and system dynamics. Such variations cause fluctuations in pressure response and flow characteristics, even under identical control commands. Therefore, it is difficult to reliably estimate resin state from hydraulic signals alone. Due to these limitations, practical indicators for quantitatively evaluating resin state are insufficient in hydraulic injection molding machines. This lack of reliable state information makes it challenging to maintain stable molding quality under varying material and operating conditions.

### 1.3. Concept of Resin State Evaluation Using AFC

As discussed in the previous subsections, directly observing resin state is difficult in injection molding processes. This difficulty is particularly severe in hydraulic injection molding machines, where reliable state estimation methods are limited. Therefore, an alternative approach is required to evaluate resin state without relying on direct measurement or detailed physical models. In this study, adaptive feedforward control (AFC) is focused on. AFC is a control method that learns repetitive characteristics of a system through repeated operations. It has been widely applied to cyclic systems, such as precision positioning and servo control, to compensate for repetitive disturbances and model uncertainties [10,11]. In conventional applications, AFC is primarily used to improve tracking performance. The learning parameters of AFC are treated as internal variables for generating feedforward compensation signals. Their physical meaning has rarely been discussed from the viewpoint of process state evaluation.

In contrast, this study adopts a different perspective on AFC. The key idea is that AFC learning parameters inherently reflect changes in system dynamics and external disturbances. In injection molding processes, such changes are strongly influenced by variations in resin state during the injection phase. Based on this observation, the learning parameters of AFC are regarded not only as control variables but also as indicators of resin state. However, it should be noted that these parameters do not represent direct physical quantities of the resin state. Instead, they should be interpreted as indicators reflecting variations in the overall process dynamics, which are influenced by multiple factors including resin properties and operating conditions. By analyzing the converged values of these parameters, resin state variations can be evaluated indirectly. This approach does not require direct measurement of resin properties or explicit physical modeling of the molding process. Furthermore, the injection phase is inherently short, which limits the learning effect within a single shot. To address this issue, a shot-to-shot compensation mechanism is introduced to accumulate learning results across successive injection cycles. This mechanism enables stable convergence of AFC learning parameters under continuous molding operation. In the experimental study presented in Section 3, the relationship between AFC learning parameters and molded products is investigated. Experiments are conducted by changing material conditions and cylinder temperature in a hydraulic injection molding machine. The results demonstrate that agreement of AFC learning parameters corresponds to agreement of molded product appearance. Through these experiments, the feasibility of using AFC learning parameters as practical indicators for aligning resin states is verified. In injection molding processes, variations in resin properties and operating conditions directly affect process stability and final product quality. The proposed AFC-based approach enables monitoring of these variations through learning parameters, providing a practical tool for improving process stability and ensuring consistent product quality. This perspective highlights the relevance of the present study to polymer processing and manufacturing.

Recent advances in injection molding control have focused on data-driven and intelligent approaches. Machine learning-based methods have been applied to process control and quality prediction [12,13,14], enabling adaptive adjustment of molding conditions based on large datasets. In addition, reinforcement learning approaches have been proposed for injection speed control in hydraulic systems [15]. Sensor-based methods have also been widely studied, particularly those utilizing cavity pressure measurements to monitor process conditions and improve product quality [16]. These approaches often rely on in-line or on-line measurements and explicit physical interpretation of process variables, such as viscosity estimation based on pressure signals. While these methods have demonstrated effectiveness, they typically require additional sensors, extensive data, or complex modeling. In contrast, the proposed AFC-based framework provides a simple and interpretable alternative by utilizing learning parameters derived from control performance. These parameters reflect variations in system dynamics influenced by process conditions, enabling indirect monitoring of resin state without requiring explicit physical modeling or additional sensors.

The novelty of this study lies in two aspects. First, AFC is extended from a control method to a state indicator by interpreting its learning parameters as representations of process dynamics. Second, a shot-to-shot compensation mechanism is introduced to enable stable learning under short-duration injection processes.

## 2. Hydraulic Injection Molding Machine and Control System

This section provides an overview of the hydraulic injection molding machine and its control system used in this study.

### 2.1. Machine Overview

**Figure 1:**
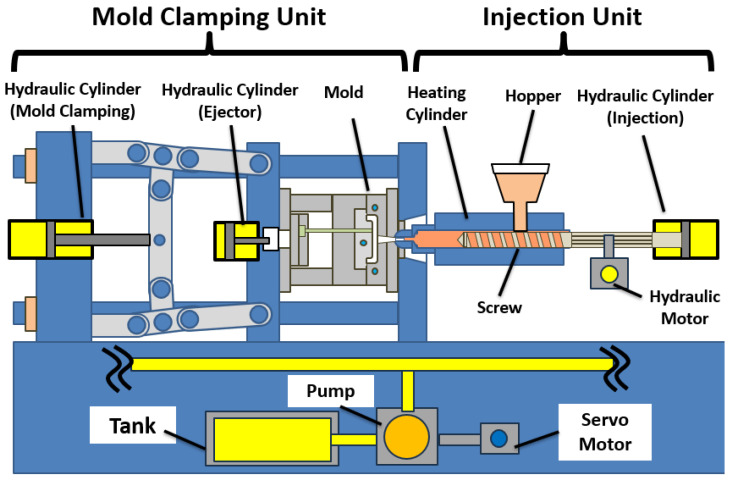
Overview of the hydraulic injection molding machine used in this study.

A hydraulic injection molding machine is a manufacturing system used to produce plastic products [17]. Molten resin is injected into a mold cavity. The resin is then cooled and solidified to form the final product. Figure 1 shows an overview of the machine structure. The machine mainly consists of a mold clamping unit and an injection unit. In this study, a variable-displacement hydraulic pump driven by a servomotor is used as the hydraulic power source. The pump draws hydraulic oil from a tank. It supplies pressurized oil to the system. The flow rate and pressure are adjusted by controlling the pump operation. Therefore, hydraulic actuators are controlled through the servomotor that drives the pump. The supplied oil is distributed to each section of the machine. It drives both rotational and translational motions. Solenoid valves are installed between the pump and each section. By switching the hydraulic circuit connections using these valves, various motions required for molding operations are realized. This configuration enables flexible control of hydraulic flow rate and pressure. As a result, precise injection motion can be achieved.

### 2.2. Mold Clamping Unit

The mold clamping unit is responsible for opening and closing the mold. It also maintains sufficient clamping force during the injection and packing phases. The movable platen is driven by a hydraulic cylinder. The generated clamping force prevents resin leakage during injection. This force is essential for maintaining dimensional accuracy of the molded product. The mold clamping unit is also equipped with an ejection mechanism. This mechanism removes the molded product from the mold after cooling. These motions are also driven by a hydraulic cylinder. Although the mold clamping unit plays an essential role in maintaining product quality, it is not directly involved in the control problem addressed in this study.

### 2.3. Injection Unit

The injection unit melts the resin and injects it into the mold cavity. Pellet resin is supplied from a hopper into a heated barrel. The resin is melted by external heaters. It is mixed by the rotational motion of the screw. During this process, molten resin accumulates in front of the screw. During the injection phase, the screw is pushed forward by a hydraulic cylinder. The screw performs translational motion. This motion injects the molten resin into the mold cavity. During the packing phase, hydraulic pressure is maintained for a certain period. This compensates for resin shrinkage. Thus, the screw performs both rotational and translational motions. Both motions are realized by hydraulic actuation.

**Figure 2:**
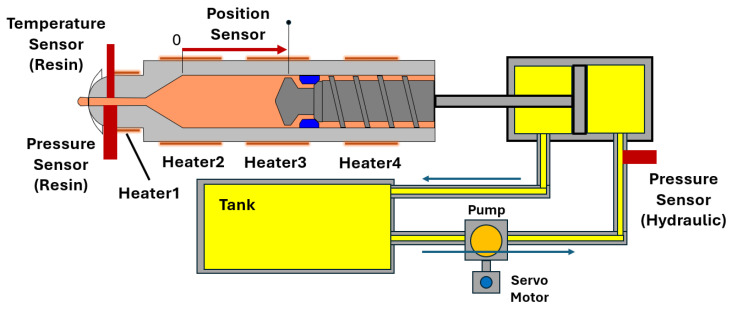
Injection-phase hydraulic circuit and sensor configuration.

In this study, the injection phase is the main focus. Figure 2 shows the hydraulic circuit and sensor arrangement during the injection phase. The translational position of the screw is measured by a position sensor. A hydraulic pressure sensor is installed on the hydraulic cylinder. This sensor can indirectly estimate resin pressure. However, as discussed in Section 1.2, accurate estimation is difficult due to variations in hydraulic characteristics.

To further monitor the injection process, additional sensors are installed near the nozzle. These include a resin pressure sensor and a resin temperature sensor. These sensors are not used for direct control. They are used for verification and evaluation.

### 2.4. Molding Process Flow

The injection molding process consists of several sequential phases. These phases are repeated cyclically during continuous operation. The overall process flow is summarized below.

(1) *Plasticizing:* Resin is melted and prepared inside the barrel.

(2) *Injection:* Molten resin is injected into the mold cavity. The screw velocity in this phase strongly affects product quality. In this study, this phase is the main focus.

(3) *Packing:* Pressure is maintained to compensate for resin shrinkage.

(4) *Cooling:* The resin solidifies inside the mold.

(5) *Mold opening and ejection:* The mold is opened and the molded product is removed.

### 2.5. Control System

The injection molding machine is controlled using a software-based programmable logic controller (PLC) environment. The software PLC allows flexible implementation of control algorithms on a general-purpose computing platform. In addition, real-time execution is ensured by a dedicated runtime system.

**Figure 3:**
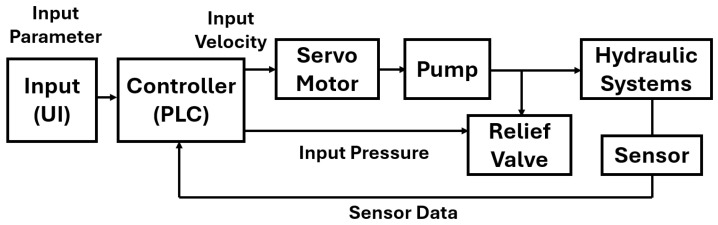
Schematic image of the injection velocity control system using PLC.

Figure 3 shows the control system structure used for injection velocity control. Sensor signals from the hydraulic system are fed back to the PLC. The screw velocity is calculated from the screw position measured by the position sensor described in Section 2.3. The computed control input is applied to the servomotor driving the hydraulic pump. By controlling the pump operation, the hydraulic flow supplied to the injection unit is regulated.

The entire control loop is executed cyclically with a fixed sampling period [18]. This configuration ensures real-time operation during continuous molding. In this study, the sampling period is set to $2.0\times 10^{-3}$ s.

## 3. Proposed Injection Velocity Control System and AFC Operation

This section describes the proposed control method based on adaptive feedforward control (AFC). AFC is introduced as a feedforward controller that learns repetitive characteristics of the injection process through repeated molding cycles. While AFC is originally designed as a control method to improve tracking performance, this study focuses on the learning parameters updated in the AFC algorithm. These learning parameters are treated not only as control variables but also as indicators that reflect variations in the internal resin state during injection.

### 3.1. Adaptive Feedforward Control (AFC)

Adaptive feedforward control (AFC) is a control method that improves tracking performance by learning repetitive characteristics of a system through repeated operations. In cyclic systems, similar reference trajectories and disturbance patterns appear in each operation cycle. AFC utilizes this property to generate feedforward compensation signals based on tracking errors observed in previous cycles. In this study, the learning parameters of AFC are treated as indicators of system dynamics. In injection molding processes, system dynamics are strongly influenced by the internal state of molten resin during the injection phase. Therefore, the AFC learning parameters reflect resin-dependent dynamic behavior. In this study, AFC is applied to injection velocity control in a hydraulic injection molding machine. The learning parameters are updated using the tracking error between the reference injection velocity and the measured screw velocity. Through repeated injection cycles, the parameters converge to values corresponding to the current molding condition. These parameters are not intended to represent direct physical measurements but rather to reflect system-level variations influenced by multiple factors. By analyzing the converged parameters, variations in resin state can be evaluated indirectly. This evaluation does not rely on direct measurement or explicit physical modeling.

Based on this concept, AFC serves two roles in this study. It improves injection velocity tracking as a feedforward controller. At the same time, its learning parameters are used as practical indicators of resin state during the injection process.

### 3.2. Theory

**Figure 4:**
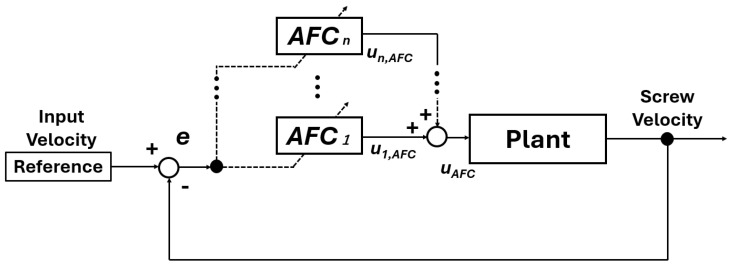
Block diagram of proposed velocity control system with AFC.

Figure 4 shows the control structure adopted in this study. The injection velocity is controlled using adaptive feedforward control. The reference injection velocity is compared with the measured screw velocity, and the tracking error is defined as e(k). Here, *k* denotes the discrete-time index corresponding to the sampling instant during the injection process.

In AFC, the feedforward input is constructed as a superposition of sinusoidal components. Each component is designed to compensate for dynamics and disturbances at a specific frequency. The output of a single AFC component is expressed as

(1) $$\begin{array}{c} u_{i}\left(k\right)=p_{i}\left(k\right)\cos\left(\omega_{i}Tk\right)+q_{i}\left(k\right)\sin\left(\omega_{i}Tk\right). \end{array}$$

Here, $p_{i}\left(k\right)$ and $q_{i}\left(k\right)$ represent the adaptive amplitudes of the cosine and sine components, respectively. The parameter $\omega_{i}$ denotes the target angular frequency, and *T* is the sampling period of the control system. The parameter *i* denotes the index of the AFC component assigned to the angular frequency $\omega_{i}$,

The damping term acts as a forgetting factor that suppresses divergence of the learning parameters and attenuates past learning effects. With this structure, each AFC component can be interpreted as a discrete-time resonant filter, providing a meaningful representation of its role in the control system.

The update laws of the learning parameters are given by

(2) $$\begin{array}{c} p_{i}\left(k\right)=e^{-\zeta_{i}\omega_{i}T}p_{i}\left(k-1\right)+\lambda_{i}e\left(k\right)\cos\left(\omega_{i}Tk+\theta_{i}\right), \end{array}$$

(3) $$\begin{array}{c} q_{i}\left(k\right)=e^{-\zeta_{i}\omega_{i}T}q_{i}\left(k-1\right)+\lambda_{i}e\left(k\right)\sin\left(\omega_{i}Tk+\theta_{i}\right). \end{array}$$

Here, $\zeta_{i}$ is the damping coefficient, $\lambda_{i}$ is the learning gain, and $\theta_{i}$ is the phase parameter. The learning gains $\lambda_{i}$ were selected to ensure stable convergence of the learning process while avoiding excessive amplification of noise. The gains were tuned so that the learning parameters converge smoothly without oscillation or divergence under the tested conditions. Unless otherwise specified, the initial values are set to

(4) $$p_{i}(0)=0,$$

(5) $$q_{i}(0)=0.$$

By superposing multiple AFC components with different angular frequencies, the AFC input is constructed as a superposition of multiple frequency components. This structure enables compensation of system dynamics distributed over a wide frequency range.

In the injection molding process considered in this study, the system dynamics during the injection phase depend on resin properties. Changes in resin viscosity and flow behavior modify the load acting on the screw and affect the system response. As a result, the AFC learning parameters converge to different values depending on the resin state. Based on this property, the AFC learning parameters are interpreted as indicators of resin state in this study.

### 3.3. Shot-to-Shot Compensation for Short Injection Phases

In injection molding processes, the duration of the injection phase is inherently short. As a result, the learning effect obtained within a single injection shot is insufficient for the AFC learning parameters to fully converge. This limitation is a practical constraint in industrial injection molding machines operated under continuous production conditions.

To address this issue, a shot-to-shot compensation mechanism is introduced. This mechanism accumulates the learning results obtained within each injection shot and transfers them to subsequent shots. By doing so, AFC learning can be extended beyond a single shot without modifying the within-shot learning structure.

Let $p_{\mathrm{acc}}$ and $q_{\mathrm{acc}}$ denote the accumulated AFC learning parameters obtained from previous shots. For the first injection shot (n=1), these accumulated parameters are initialized as

(6) $$p_{i,\mathrm{acc}}=0,$$

(7) $$q_{i,\mathrm{acc}}=0.$$

During a single injection shot, the AFC compensation signal is constructed as

(8) $$\begin{array}{cc} u_{i}\left(k\right) & =\left(p_{i,\mathrm{acc}}+p_{i}\left(k\right)\right)\cos\left(\omega_{i}Tk\right)\\ \; & \;+\left(q_{i,\mathrm{acc}}+q_{i}\left(k\right)\right)\sin\left(\omega_{i}Tk\right). \end{array}$$

Here, the symbol $k_{\mathrm{end}}$ represents the final time step of the injection phase. Accordingly, $p_{i}\left(k_{\mathrm{end}}\right)$ and $q_{i}\left(k_{\mathrm{end}}\right)$ correspond to the final learning results obtained during that shot.

After completion of the *n*-th injection shot, the accumulated parameters are updated as follows:

(9) $$p_{i,\mathrm{acc}}\leftarrow p_{i,\mathrm{acc}}+p_{i}(k_{\mathrm{end}}),$$

(10) $$q_{i,\mathrm{acc}}\leftarrow q_{i,\mathrm{acc}}+q_{i}(k_{\mathrm{end}}).$$

The updated accumulated parameters are then used as the initial compensation values for the subsequent shot.

In continuous injection molding operations, the converged values of the AFC learning parameters obtained in each shot are not necessarily identical. Small variations in operating conditions, such as thermal fluctuations and transient flow behavior, cause shot-to-shot variations in the learning results even under nominally identical settings. To prevent unstable accumulation caused by excessive variation in the learning results, a threshold-based update rule is introduced. Let $J(k_{\mathrm{end}})$ denote an evaluation index calculated from the tracking error during a single injection shot. The accumulated parameters are updated only when the variation remains within an acceptable range:

(11) $$\begin{array}{c} J\left(k_{\mathrm{end}}\right)<J_{\mathrm{th}}, \end{array}$$

where $J_{\mathrm{th}}$ is a predefined threshold. Here, the evaluation index $J(k_{\mathrm{end}})$ is defined as the magnitude of the AFC learning parameter at the final time step of the injection phase. The evaluation is performed over the injection phase, and the threshold is applied to the value at $k=k_{\mathrm{end}}$. In this study, $J(k_{\mathrm{end}})$ is treated as a dimensionless quantity. The threshold value $J_{\mathrm{th}}$ was determined empirically based on experimental observations to distinguish meaningful parameter updates from noise and to prevent unstable accumulation. The selected value was found to provide stable convergence behavior under the tested conditions. If this condition is not satisfied, the accumulated parameters are not updated for that shot:

(12) $$p_{i,\mathrm{acc}}\leftarrow p_{i,\mathrm{acc}},$$

(13) $$q_{i,\mathrm{acc}}\leftarrow q_{i,\mathrm{acc}}.$$

This update strategy suppresses the influence of shot-to-shot variability and enables stable accumulation of AFC learning parameters toward representative values for the current molding condition. Although the accumulation is implemented as a simple summation, potential drift due to low-frequency bias or abnormal shots is mitigated by the threshold-based update rule. As observed in the experimental results, the learning parameters converge to stable values under the tested conditions. Further improvements, such as introducing forgetting factors or robust statistical filtering, are considered as future work.

By repeating this update process, the AFC system achieves learning on two time scales. Fast adaptation occurs within each injection shot through $p_{i}\left(k\right)$ and $q_{i}\left(k\right)$. Slow accumulation across repeated shots is achieved through $p_{i,\mathrm{acc}}$ and $q_{i,\mathrm{acc}}$. This two-time-scale learning structure enables stable convergence of AFC learning parameters under short-duration injection phases. In practical applications, the accumulated parameters should be reset or re-initialized when significant changes in process conditions occur, such as material switching, temperature variation, or abnormal operating states. In this study, the experiments were conducted under controlled conditions, and therefore reset operations were not required during the evaluation.

The AFC generates control inputs to compensate for variations in system dynamics, and the learned coefficients $p_{i}\left(k\right)$ and $q_{i}\left(k\right)$ determine the amplitude of the sinusoidal components at each frequency. Therefore, the magnitude of these coefficients represents the required compensation level. In injection molding processes, this required compensation is influenced by variations in system load associated with resin viscosity and flow behavior. As a result, changes in resin state are reflected in the magnitude of the learned parameters $p_{i}\left(k\right)$ and $q_{i}\left(k\right)$ through their effect on the system dynamics.

In this study, multiple AFC components with different angular frequencies are implemented in parallel. The overall AFC compensation input is expressed as

(14) $$\begin{array}{c} u_{\mathrm{AFC}}\left(k\right)=\sum_{i=1}^{N}u_{i}\left(k\right). \end{array}$$

The parameter *N* denotes the total number of AFC components. Through this formulation, the AFC system preserves learning results across shots while maintaining adaptability within each injection cycle.

### 3.4. Experimental Conditions

This subsection summarizes the experimental conditions and confirms the basic operation of the proposed AFC framework. The main molding conditions and AFC parameters used in this study are summarized in Table 1. In this study, the focus is placed on the injection phase. Therefore, the packing/holding phase is omitted, and the corresponding parameters are not applied.

**Figure 5:**
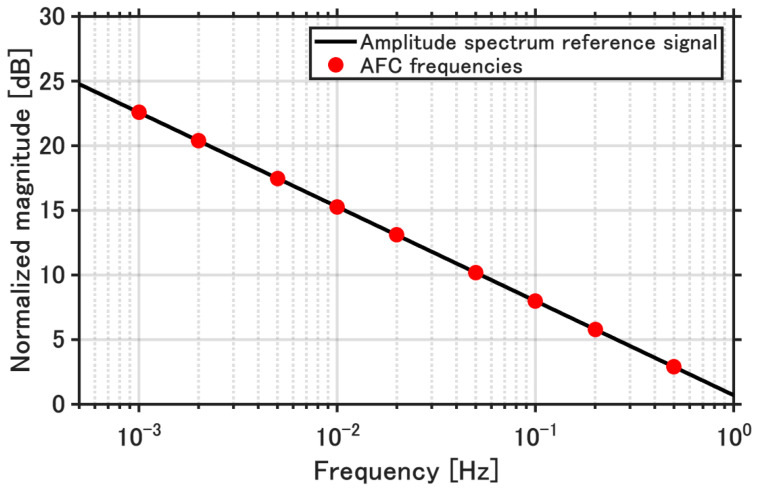
Normalized frequency spectrum of the reference model output and selected AFC frequencies.

The AFC frequency parameters were determined based on the frequency content of the reference model output. The reference model is a first-order system identified from measured data of the actual injection molding machine. The output response of this reference model under step excitation was transformed into the frequency domain. The analysis results and the selected AFC frequencies are illustrated in Figure 5. The spectrum was normalized by its maximum component, so that the dominant frequency range could be clearly identified. In this analysis, the DC component was excluded, which emphasizes transient and fluctuation components relevant to the AFC operation. The AFC frequencies were selected to cover the dominant frequency range observed in the normalized spectrum.

**Figure 6:**
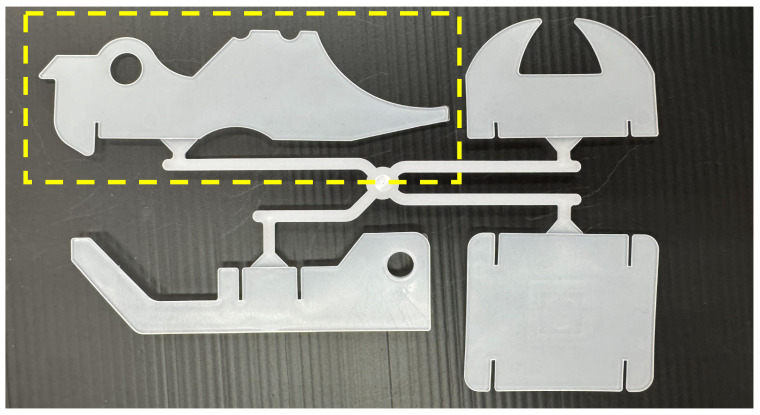
Molded product used in the experiments.

The heater positions correspond to the numbered locations shown in Figure 2. The molded product used in this study is shown in Figure 6. In this study, the upper-left product shown in Figure 6 was used for evaluation. All experiments were conducted using this product geometry in order to maintain consistent flow and load conditions during the injection phase.

In this study, multiple AFC components with different angular frequencies are implemented in parallel. The target angular frequencies are selected based on the dominant dynamic characteristics observed during the injection phase. These frequencies are fixed throughout the experiments unless otherwise specified. Other molding conditions, such as the injection velocity profile and cylinder temperature, are kept constant in order to isolate the effect of resin properties.

**Figure 7:**
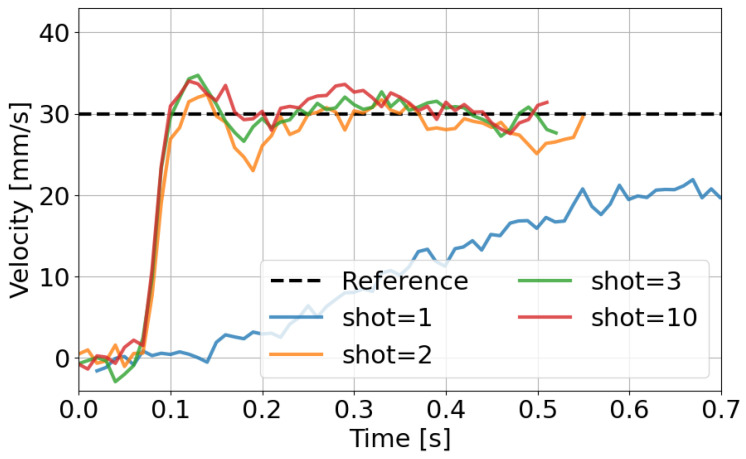
Injection velocity responses at different shot numbers with AFC. The dashed line indicates the reference velocity, and the solid lines represent measured screw velocity at each shot. Tracking performance improves as the number of shots increases.

**Figure 8:**
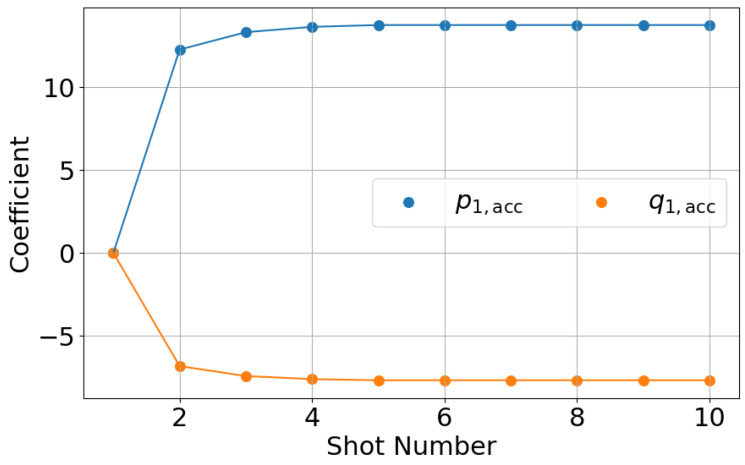
Shot-to-shot transition of accumulated AFC learning parameters. The parameters increase during initial shots and converge to steady values, indicating stable learning behavior.

Figure 7 and Figure 8 verify the fundamental behavior of the AFC system under the conditions listed in Table 1. Figure 7 shows the injection velocity responses at different shot numbers. In the initial shots, the tracking performance is limited due to the absence of accumulated learning. The distinct behavior observed at shot = 1 is attributed to the initial condition of the AFC learning parameters. Since no prior learning is available at the first shot, the compensation capability is limited. As the learning parameters are updated over successive shots, the tracking performance improves.

Figure 8 shows the shot-to-shot transition of the accumulated AFC learning parameters. The learning parameters increase during the early shots and then converge to steady values. This behavior confirms that the proposed shot-to-shot compensation mechanism functions as intended. Based on these results, it is confirmed that the AFC system achieves stable learning and consistent injection velocity control under the experimental conditions summarized in Table 1.

### 3.5. Comparison with PID Control

To evaluate the effectiveness of the proposed AFC method, a comparison with conventional PID control is conducted.

All experiments were performed under identical molding conditions to ensure a fair comparison. The injection velocity profile, cylinder temperature, and other operating conditions were kept constant throughout the experiments. In addition, the controller parameters for both PID and AFC were fixed for all step conditions, and no step-dependent tuning was applied. For the AFC method, the responses shown correspond to the steady-state condition after the learning parameters have converged. Each waveform represents the average of five consecutive shots to reduce the influence of shot-to-shot variability.

**Figure 9:**
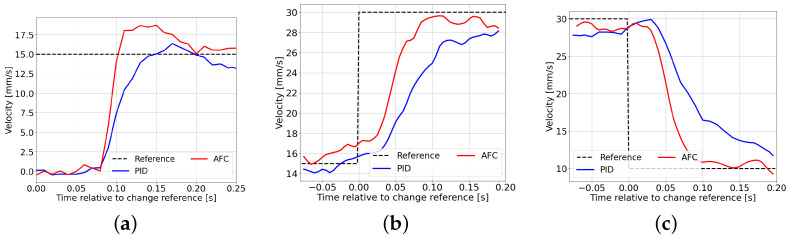
Comparison of injection velocity responses between PID control and AFC for multiple step changes in the reference signal. The dashed line represents the reference velocity, while the solid lines indicate the measured responses. (**a**) Step 1: increase from 0 to 15 mm/s. (**b**) Step 2: increase from 15 to 30 mm/s. (**c**) Step 3: decrease from 30 to 10 mm/s.

Figure 9 shows the injection velocity responses for multiple step changes in the reference signal. The dashed line represents the reference velocity, while the solid lines indicate the measured responses.

The results demonstrate that AFC achieves faster response and reduced tracking delay compared to PID control for all step conditions. In particular, AFC shows improved performance in both acceleration and deceleration phases, which are critical in multi-stage injection processes.

Furthermore, the tracking performance is evaluated using the root mean square error (RMSE) between the reference and measured injection velocities. The RMSE values are calculated based on the averaged waveform over five consecutive shots. The RMSE is defined as follows:

(15) $$\mathrm{RMSE}=\sqrt{\frac{1}{N}\sum_{k=1}^{N}{\left(r(k)-y(k)\right)}^{2}}$$

where r(k) and y(k) denote the reference and measured injection velocities, respectively, and *N* is the number of samples.

The calculated RMSE values for each condition are as follows:

- Step1: PID = 9.3461, AFC = 9.1034 (2.6% improvement)
- Step2: PID = 7.0333, AFC = 5.1972 (26.1% improvement)
- Step3: PID = 10.1144, AFC = 8.1988 (18.9% improvement)

It should be noted that the RMSE at the first step is nearly identical for PID and AFC. This indicates that the PID controller is appropriately tuned and provides a reasonable baseline performance. Therefore, the observed improvements in AFC are not due to insufficient PID tuning but are attributed to the enhanced control capability of the proposed method, particularly during reference changes.

These results indicate that the proposed AFC method provides superior tracking performance under identical conditions, demonstrating its effectiveness for multi-stage injection velocity control.

## 4. Resin State Observation and Alignment Using AFC Learning Parameters

This section presents experimental validation of the proposed AFC-based framework. The purpose of this section is to demonstrate the use of AFC learning parameters as indicators of resin state. Their applicability is examined in a hydraulic injection molding machine.

In electric injection molding machines, resin state can be indirectly inferred. Such information can be reflected in control and molding conditions. In hydraulic injection molding machines, this approach is difficult. In this study, AFC learning parameters are interpreted as state-related variables. They are obtained from injection velocity tracking behavior. They do not rely on explicit physical models. If AFC learning parameters represent resin state, their alignment should indicate state alignment. This hypothesis is examined through experiments.

Two kinds of experiments are conducted in this section. In the first experiment, the material is changed under identical molding conditions. The response of AFC learning parameters is analyzed. In the second experiment, molding conditions are adjusted. The AFC learning parameters are guided to reference values. Through these experiments, the role of AFC learning parameters is clarified. Their applicability as resin state indicators is demonstrated.

### 4.1. Experimental Conditions and Evaluation Metrics

This subsection summarizes the experimental conditions and evaluation methods used in this study. Unless otherwise stated, the same conditions are applied to all experiments. Two polypropylene materials are used in the experiments. PP707 is used as the reference material. PP705 is used as the comparison material. The material properties of the polypropylene materials used in this study are summarized in Table 2. The melt flow rate (MFR) and deflection temperature under load (DTUL) were obtained from the manufacturer’s datasheet and used to characterize the flowability and thermal properties of the materials. As shown in Table 2, PP707 exhibits a higher MFR than PP705, indicating lower viscosity and higher flowability. These differences in material properties are expected to influence the resin flow behavior during the injection process. These properties provide a physical basis for interpreting differences in AFC learning parameters.

The injection velocity profile is fixed throughout all experiments. Cylinder temperature is set to predefined values depending on the experiment. The same mold as shown in Figure 6 of Section 3 is used in all experiments. Other molding conditions are kept constant to clarify the effect of resin state. AFC learning parameters are influenced by multiple factors, such as hydraulic characteristics, friction, and thermal conditions. In this study, these factors were kept as constant as possible; therefore, the observed variations are mainly attributed to differences in material properties.

The number of AFC components and their frequency settings are identical to those used in Section 3. AFC learning parameters are used as the primary evaluation variables. Their shot-to-shot transitions and converged values are analyzed. Molded product appearance is used as an evaluation result. Molded product appearance is presented only for regions where notable changes are observed in the experiments. Resin pressure measured by the pressure sensor shown in Figure 2 is also used for evaluation.

### 4.2. Experiment 1: Effect of Material Change on AFC Learning Parameters

This experiment investigates the effect of material change on AFC learning parameters. The objective is to confirm that the learning parameters reflect differences in resin state.

Continuous molding is performed while changing the material from PP707 to PP705. Molding is continued until the material in the system is completely replaced by PP705. The total number of shots required for material replacement is 70. All molding conditions except the material are kept constant. The injection velocity profile and cylinder temperature are unchanged. AFC learning is executed continuously using the shot-to-shot compensation scheme.

**Figure 10:**
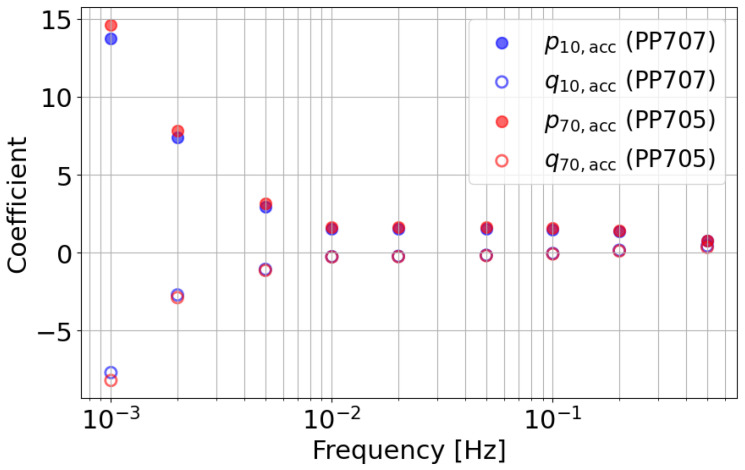
Distribution of accumulated AFC learning parameters at different frequencies. The magnitude of parameters differs between materials, while the frequency-dependent distribution shows similar trends.

Figure 10 shows the distributions of the accumulated AFC learning parameters at nine target frequencies for Shot 10 and Shot 70. Shot 10 corresponds to the condition using PP707, while Shot 70 corresponds to the condition using PP705. For both *p* and *q*, the absolute values of the parameters differ between the two shots. In contrast, the frequency-dependent distribution exhibits a similar tendency in both cases. Since PP705 has a higher viscosity than PP707, the overall increase in the absolute parameter values is reasonable. This result indicates that the relative contribution of each frequency component is preserved. At the same time, the overall parameter level reflects material-dependent differences in resin state. Based on this observation, the following discussion focuses on the *p* parameter of the lowest-frequency AFC component (i=1).

**Figure 11:**
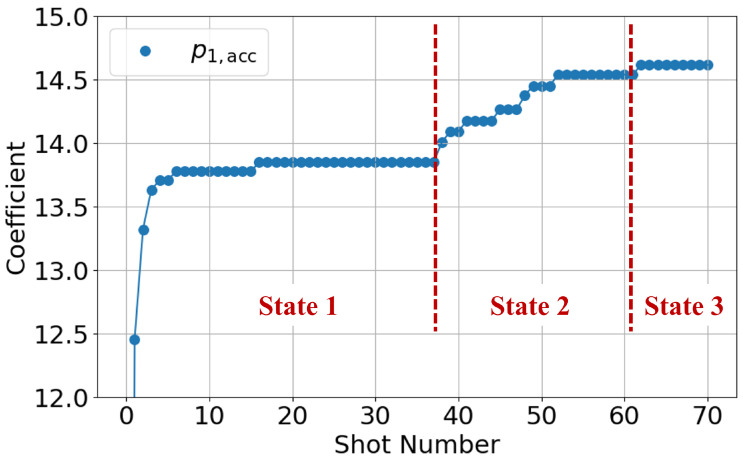
Shot-to-shot transition of the accumulated AFC learning parameter $p_{i,\mathrm{acc}}$ (i=1). Three distinct states are observed, corresponding to material conditions during the replacement process.

Figure 11 shows the shot-to-shot transition of the accumulated *p* parameter for the AFC component (i=1) from Shot 1 to Shot 70. Since the accumulated value is zero at the first shot, the figure highlights the data from the second shot onward. Three distinct states can be identified from the transition.

In State 1 (Shots 1–37), the parameter remains around a constant value of approximately 13.8. In State 2 (Shots 38–61), the parameter exhibits a rapid increase. In State 3 (Shots 62 onward), the parameter converges to a stable value of approximately 14.6.

State 1 corresponds to the condition where only PP707 remains inside the cylinder. Even after new material is supplied to the hopper, the original material remains in the cylinder for several shots. Therefore, no material change occurs during this state. Consistently, no change in molded products is observed in State 1. State 2 corresponds to a mixed condition of PP707 and PP705. During material replacement, molten materials coexist inside the cylinder. State 3 corresponds to the condition where only PP705 remains.

**Figure 12:**
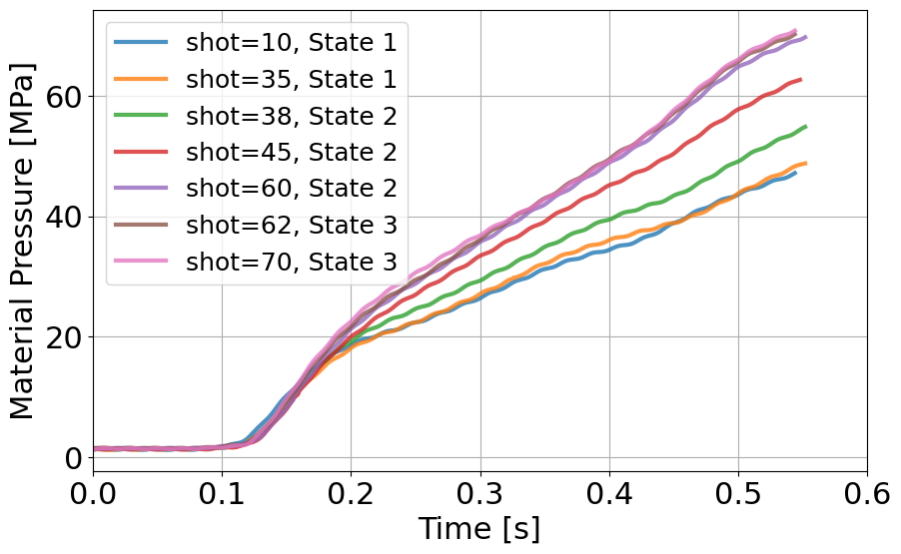
Resin pressure responses during material replacement. An increase in pressure level is observed as material viscosity increases.

**Figure 13:**
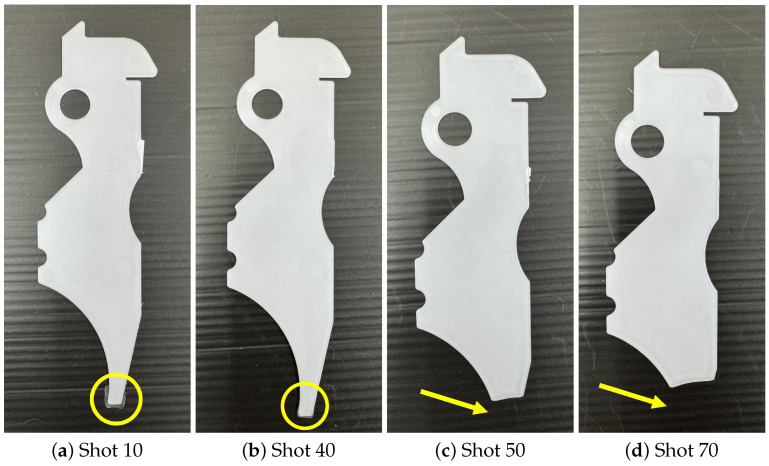
Molded products obtained at representative shot numbers during material replacement. Changes in product appearance correspond to transitions in AFC learning parameters.

To verify these interpretations, resin pressure and molded product appearance are evaluated in Figure 12 and Figure 13. Figure 12 shows that resin pressure waveforms remain unchanged in State 1. In State 2, the pressure level increases. In State 3, the waveform becomes stable again. These trends are consistent with those of the AFC learning parameters. Also, an increase in resin pressure corresponds to an increase in resin viscosity. Since the AFC learning parameters exhibit similar trends, they can be regarded as indirectly representing resin state.

Figure 13 shows the molded products obtained at representative shots. In State 2, a rapid change in product appearance is observed. At Shot 10, excess material is observed at the lower part of the product. At Shot 40, where the AFC parameters begin to change, this excess disappears. At Shots 50 and 70, further reduction in product size is observed. These appearance changes correspond well to the transition of AFC learning parameters.

From these results, it is confirmed that AFC learning parameters change in response to material replacement. The changes reflect material-dependent resin state differences under identical molding conditions. Therefore, AFC learning parameters can be regarded as indicators of resin state.

### 4.3. Experiment 2: Alignment of Resin State Using AFC Learning Parameters

This experiment investigates whether alignment of AFC learning parameters leads to alignment of resin state. The objective is to demonstrate that resin state can be indirectly reflected in molding conditions using AFC learning parameters. As shown in Experiment 1, AFC learning parameters converge to different steady values for PP707 and PP705 under identical molding conditions. It is known that the viscosity of polypropylene decreases as temperature increases [7]. Therefore, this experiment aims to increase the resin temperature of PP705 to achieve a viscosity state equivalent to that of PP707. The converged value obtained for PP707 is used as the reference value. Based on this reference, PP705 is molded while changing the cylinder temperature. The injection velocity profile and other molding conditions are unchanged.

During continuous molding with PP705, the cylinder temperature is adjusted. This adjustment is performed to guide the AFC learning parameters from the initial steady value of PP705 toward the reference value of PP707. AFC learning is executed continuously using the shot-to-shot compensation scheme.

**Figure 14:**
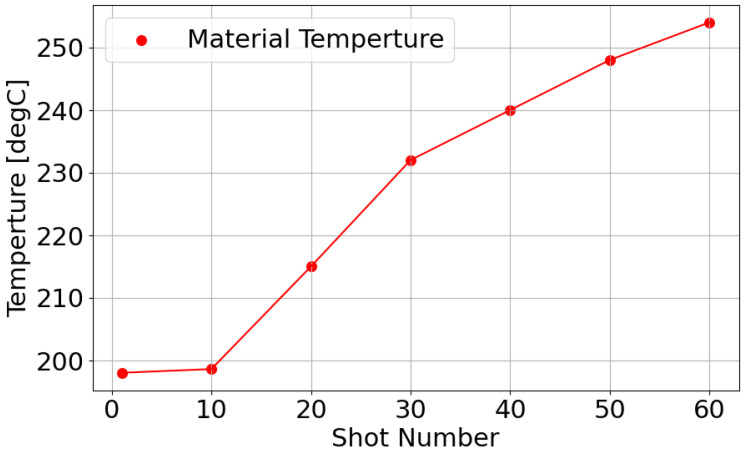
Resin temperature profile during the temperature increase experiment.

Figure 14 shows the resin temperature measured every ten shots in this experiment. The resin temperature is measured using the nozzle temperature sensor shown in Figure 2. As molding proceeds, the resin temperature increases stepwise. This temperature adjustment is applied to align the AFC learning parameters with the reference value obtained for PP707.

**Figure 15:**
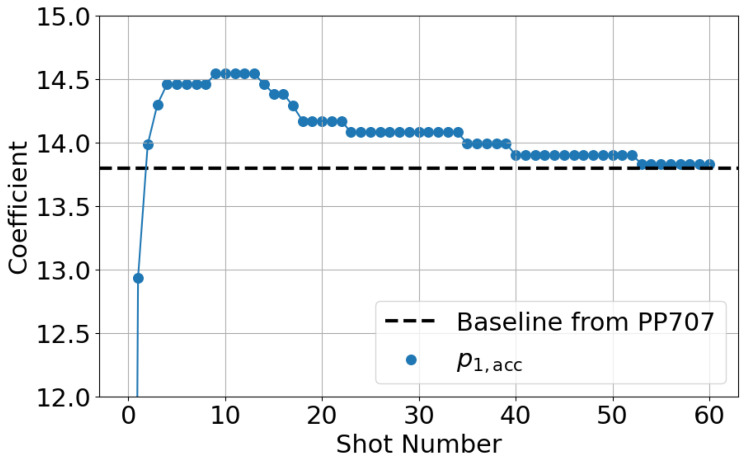
Shot-to-shot transition of the AFC learning parameter during resin temperature increase. The parameter converges toward the reference value as the temperature increases.

Figure 15 shows the shot-to-shot transition of the accumulated *p* parameter for the AFC component (i=1). Under the initial temperature condition, the parameter converges to the steady value obtained for PP705 in Experiment 1. As the resin temperature increases, the parameter changes stepwise. Finally, the parameter converges to a value close to the PP707 reference value.

**Figure 16:**
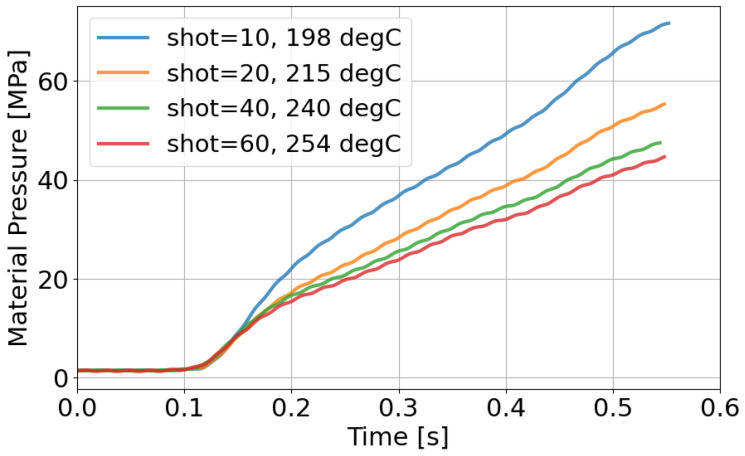
Resin pressure responses during the temperature increase experiment. Pressure decreases as resin temperature increases, indicating reduced viscosity.

Figure 16 shows the resin pressure responses to representative shots. As the resin temperature increases, the pressure level decreases. This decrease corresponds to a reduction in resin viscosity. When the AFC learning parameter remains at the PP705 steady value, the pressure waveform differs from that of PP707. When the AFC learning parameter converges to the reference value, the pressure waveform becomes similar. This result indicates alignment of resin flow behavior.

**Figure 17:**
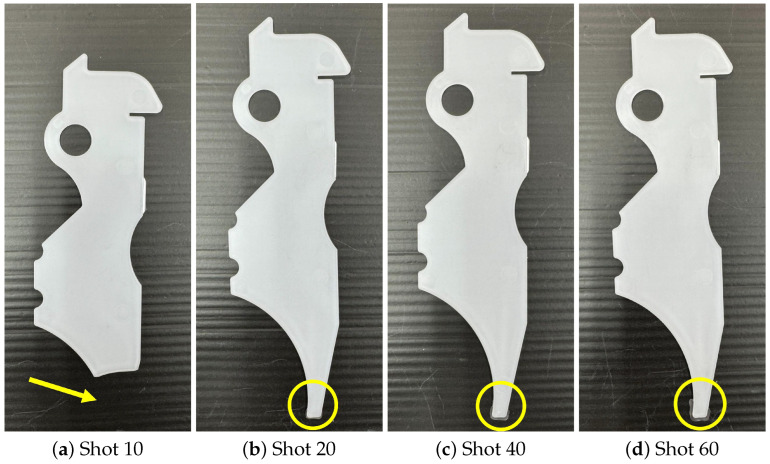
Molded products obtained with representative shots during the temperature increase experiment. Product appearance becomes similar to the reference condition as AFC learning parameters converge.

Figure 17 shows the molded products obtained with representative shots. For comparison, the molded product obtained under the reference condition in Experiment 1, shown in Figure 13a, is used. When the AFC learning parameter differs from the reference value, clear differences in product appearance are observed compared with Figure 13a. When the AFC learning parameter approaches the PP707 reference value, the molded product appearance becomes similar to that shown in Figure 13a.

From these results, alignment of AFC learning parameters leads to alignment of resin state. Therefore, AFC learning parameters are effective indicators for aligning resin state through cylinder temperature adjustment. This approach enables adjustment of resin state-based conditions in hydraulic injection molding machines.

### 4.4. Section Summary

In this section, experimental investigations on resin state observation and alignment using AFC learning parameters are presented. Experiment 1 demonstrates that AFC learning parameters converge to different steady values depending on the molding material under identical conditions. The results indicate that AFC learning parameters reflect material-dependent resin state differences.

Experiment 2 demonstrates that adjusting cylinder temperature to align with AFC learning parameters also results in alignent with resin state. When the AFC learning parameters converge to the reference value obtained for PP707, resin pressure behavior and the molded product’s appearance become similar. These results confirm that AFC learning parameters can be used as effective indicators for indirect observation and alignment of resin state in hydraulic injection molding machines. It should be noted that the experimental results presented in this study are primarily demonstrative. A more rigorous statistical validation based on repeated experiments and quantitative variability analysis would further strengthen the conclusions and is considered future work. AFC learning parameters can be utilized as practical indicators for adjusting molding conditions. By matching these parameters to a reference condition associated with desired product quality, it becomes possible to align process conditions across different materials or temperatures. This approach contributes to reducing operator dependency and improving reproducibility in injection molding processes.

## 5. Conclusions

This paper proposed an adaptive feedforward control (AFC)-based framework for injection velocity control in hydraulic injection molding machines. The proposed method utilizes AFC learning parameters as practical indicators of resin state. In hydraulic systems, direct observation of molten resin state is inherently difficult due to limited sensor availability and variations in hydraulic characteristics. To address this limitation, AFC was introduced as a feedforward controller whose learning parameters reflect the repetitive dynamic behavior during the injection phase. Furthermore, a shot-to-shot compensation mechanism was developed to overcome the short duration of the injection phase by accumulating learning results across cycles. This mechanism enabled stable convergence of AFC learning parameters under continuous molding operation without modifying the within-shot control structure.

Experimental results demonstrated that AFC learning parameters change systematically in response to material replacement under identical molding conditions. These parameter transitions showed strong consistency with changes in resin pressure and the molded product’s appearance, indicating that the learning parameters effectively capture material-dependent variations in resin state. Furthermore, by adjusting the cylinder temperature of a different material, the AFC learning parameters were successfully guided toward those of a reference material. Under these conditions, the molded product’s appearance also became similar, demonstrating that alignment of AFC learning parameters corresponds to alignment of resin states.

These results indicate that AFC learning parameters can serve as practical and interpretable state indicators for resin condition alignment in hydraulic injection molding processes. A key contribution of this study is the reinterpretation of AFC not only as a control method but also as a tool for process state evaluation that does not require additional sensors or explicit physical modeling. However, the experimental validation in this study was conducted under limited conditions. More rigorous statistical validation and evaluation using a wider range of materials and operating conditions are required to generalize the findings. In industrial applications, product quality is often evaluated using quantitative indicators such as part weight. Advanced industrial control systems utilize part weight as a feedback variable to stabilize molding conditions. Integrating these quantitative evaluation methods with the proposed AFC-based framework is an important direction for future work.

## Figures and Tables

**Table 1:** Experimental conditions and AFC’s frequency parameters.

Item	Value
Material (reference)	PP707
Injection velocity profile	30 mm/s
Injection pressure	80%
Measuring position	18 mm
V/P position	6 mm
Mold temperature	27 degC
Cooling time	10 s
Cylinder temperature (ch1, ch2, ch3, ch4)	[210,220,210,200] degC
Sampling period $T_{s}$	$2.0\times 10^{-3}$ s
Number of AFC components *N*	9
Target angular frequencies $\omega_{i}$ [rad/s]	2π×10−3×{1,2,5,10,20,2π×10−3×{50,100,200,500}

**Table 2:** Material properties and test conditions of polypropylene used in this study [19].

Property	Test Condition	PP707	PP705
Melt flow rate (MFR) [g/10 min]	230 °C	30	9.0
Deflection temperature under load (DTUL) [°C]	0.45 MPa	90	105

## Data Availability

The original contributions presented in this study are included in the article. Further inquiries can be directed to the corresponding author.
